# Titles of Papers, Program, Etc.

**Published:** 1892-03

**Authors:** 


*To the Members of the State Medical Association:*

Four weeks of intense suffering from la grippe has somewhat delayed the issuance of the annual circular required of me by our By-Laws. You are hereby reminded that the next, and twenty-fourth annual meeting of the Texas State Medical Association, will be held in the city of Tyler, beginning at 10 o'clock a. m. on Tuesday, April 26, and ending on Friday, April 30, 1892. Allow me to impress upon your minds the importance and necessity of a full attendance at this annual meeting, and to urge you to let no ordinary excuse prevent you from being present on this occasion. Our Association is admitted to be in a more prosperous and harmonious condition than for many years past, and the coming meeting at Tyler bids fair to have less friction and more interesting discussions than is usually the case--more to draw and interest scientific men, and to make the meeting educating and profitable. The physicians of Tyler, by prompt and early action, have already given assurance of a cordial reception, and the most hospitable entertainment during our stay in that city, and everything possible will be done by them to make our visit pleasant and enjoyable. Chairmen of Sections are especially urged to be promptly on hand, with as many and as interesting papers as can possibly be obtained. Come one—come all—and let us have a glorious good time together. Bring with you recruits to our ranks from among the worthy members of our profession, who have not yet affiliated with the State Association, and so let us swell our numbers until we shall embrace in our membership all honorable physicians within our State. Let us come together in Tyler fully imbued with the spirit of a resolution reported by the Judicial Council at our last meeting, and adopted by the Association: "That members be requested and urged to hereafter endeavor to keep out of the State Association, or its Judicial Council, all matters of a personal character," and that we all strive to cultivate and promote a spirit of unity in the bond of peace. I have the honor to be,

Yours truly,

W. H. WILKES, President.

---

Titles of papers to be read should be in the hands of the General Secretary by March 15th, for publication in the March numbers of the State medical journals.

W. H. WILKES, M. D., President,

Waco, Texas.

H. A. WEST, M. D., General Secretary,
2107 Market St., Galveston.

NOTE.—The usual reductions will be made by the railroads.

PROGRAMME (IN PART).

In pursuance of the above announcement, Secretary West requests us to publish the following list of titles to papers to be read. It is, of course, incomplete, but it is no fault of the Secretary:

SECTION ON PRACTICE OF MEDICINE.

Perityphlitis: by J. P. Tucker, M. D., Overton, Texas.

The Opium Habit: by W. B. Brooks, M. D., Dallas, Texas.

Treatment of the Morphine Habit; by John H. Pope, M. D., Marshall, Texas.

The Diagnosis of Miliary Tuberculosis: by Prof. H. A. West, M. D., Galveston, Texas.

A Case of Chronic Dysentery: by R. T. Knox, M. D., Gonzales, Texas.

Relationship between Rheumatism and Chorea: by H. P. Cooke, M. D., Galveston, Texas.

Cardiac Thrombosis: by Jas. Kennedy, M. D., San Antonio, Texas.

SECTION ON OBSTETRICS, ETC.

A Case of Sudden Death after Abortion: by Clay Johnson, M. D., Corsicana, Texas.

SECTION ON SURGERY, ETC.

Remarks on Certain Injuries of the Upper Extremity: by Prof. James E. Thompson, M. D., Galveston, Texas.

The Radical Cure of Hernia (Bassini's Method Illustrated), with Remarks on Recurrent and Ventral Hernia: by Sam'l E. Milliken, New York.

The Trained Nurse: by W. H. Baldinger, M. D., Galveston, Texas.

SECTION ON MEDICAL JURISPRUDENCE, ETC.

An Inebriate Asylum for Texas: by W. B. Kennedy, M. D., Hillsboro, Texas.

Some of the Causes of Insanity: by John Preston, M. D., Supt. State Lunatic Asylum, Terrell, Texas.

SECTION ON GYNECOLOGY.

Causes and Management of Irritable Bladder in the Female: by Prof. J. F. Y. Paine, M. D., Galveston, Texas.

The After Treatment of Patients in Abdominal Surgery: by Joseph Price, M. D., Philadelphia, Pa.

The Finger in Practice: by J. S. Poynor, M. D., Bartlett, Texas.

RAILROAD AND HOTEL RATES, ETC.

The following letter from C. A. Smith, M. D., Tyler, Texas, chairman of the Committee of Arrangements, gives the results of the work of the Arrangement Committee to March 13th, instant:

TYLER, TEXAS, March 13, 1892.

*H. A. West, M. D., Secretary T. S. M. A., Galveston, Texas:*

DEAR DOCTOR:—Letters have been sent to all the railroads in the State, asking them to put on a reduced rate for the meeting, tickets to be on sale April 24th, 25th and 26th, good to return until May 1st.

Thus far the following roads have agreed to make a rate of one and one-third fare for the round trip, or four cents per mile one way:

The St. Louis Southwestern R'y, including the Tyler Southwestern R'y; the Missouri, Kansas & Texas R'y; the Fort Worth & Denver City R'y; the East Line & Red River R'y; the Galveston, Harrisburg & San Antonio R'y, Southern Pacific route; the Gulf, Colorado & Santa Fe R'y; the Houston & Texas Central R'y; the Houston & Shreveport R'y.

There has been hardly time to hear from all the roads since the letter was sent out, but I think it safe to say that all the roads in the State will sell tickets at one and one-third fare for the round trip, or four cents per mile one way.

We have a special committee on hotels and accommodations, who have not as yet completed their arrangements, but we feel assured we will be able to take care of all who may come, at rates from $1.50 to $2.50 per day. I will advise you more fully in a short time about hotels and rates.

We have secured the use of the Grand Opera House for the convention. It is one of the finest in the State, with a seating capacity of 1500. Its acoustic properties are also very good. We will also provide room for surgical and pharmaceutical displays.

It is the intention of your local committee to send out a circular to all members of the regular profession of the State, about April 1st, showing railroad and hotel rates, and such other information as is necessary.

The local physicians here are well organized, and are all working hard to make the meeting in our city a success, and all who attend may be sure of a pleasant and profitable time.

Yours truly,

C. A. SMITH,

Chairman Committee of Arrangements, T. S. M. A.

---

A Sad Case.—Mention was made in the last issue of the JOURNAL of the killing of Dr. Cook at Liberty, Texas, by Dr. J. O. Furlow. The JOURNAL is not in possession of the facts relating to the killing, nor the causes which led to it. But we have learned that Dr. Furlow is, and for several years past has been, in very delicate health; and in consequence of certain drugs it is said that his mind is affected,—some say, to the verge of insanity. During his imprisonment he leaves an invalid young wife and four small children *in utter destitution.* Mrs. Furlow has written the JOURNAL and makes an appeal for pecuniary aid,—offering to sell the doctor's library and surgical instruments. Should any of our readers—the physicians of Texas—see proper to respond to this appeal it will be an act of real charity worthily bestowed and gratefully received. Address Mrs. Alice Furlow, Liberty, Texas.

We know nothing of the condition of the family of the dead man, but as he was an old resident physician at Liberty it is to be hoped he did not leave his family in want. How sad—that in in evil hour this man—once a promising young physician, should, by a rash act, plunge his own family and that of a brother physician into the deepest distress. Poor little children! Truly are the sins of the father visited upon them in this instance.

